# Effects of cannabidiol on risky decision-making and autonomic function in alcohol use disorder

**DOI:** 10.1093/alcalc/agag068

**Published:** 2026-10-14

**Authors:** Ellen McGuire, Tristan Hurzeler, Warren Logge, Joshua Watt, Iain S McGregor, Anastasia Suraev, Paul S Haber, Kirsten C Morley

**Affiliations:** Edith Collins Centre for Translational Research (Alcohol, Drugs & Toxicology), Royal Prince Alfred Hospital, Sydney Local Health District, Camperdown, NSW 2050, Australia; Leiden University, Faculty of Medicine, Leiden University Medical Centre, Leiden, Zuid-Holland, Albinusdreef 2, 2333 ZA Leiden, Netherlands; Edith Collins Centre for Translational Research (Alcohol, Drugs & Toxicology), Royal Prince Alfred Hospital, Sydney Local Health District, Camperdown, NSW 2050, Australia; Specialty of Addiction Medicine, Sydney Medical School, Faculty of Medicine and Health, University of Sydney, Camperdown NSW 2050, Australia; Edith Collins Centre for Translational Research (Alcohol, Drugs & Toxicology), Royal Prince Alfred Hospital, Sydney Local Health District, Camperdown, NSW 2050, Australia; Specialty of Addiction Medicine, Sydney Medical School, Faculty of Medicine and Health, University of Sydney, Camperdown NSW 2050, Australia; Edith Collins Centre for Translational Research (Alcohol, Drugs & Toxicology), Royal Prince Alfred Hospital, Sydney Local Health District, Camperdown, NSW 2050, Australia; Specialty of Addiction Medicine, Sydney Medical School, Faculty of Medicine and Health, University of Sydney, Camperdown NSW 2050, Australia; Lambert Initiative for Cannabinoid Therapeutics, University of Sydney, Camperdown NSW 2050, Australia; Faculty of Science, School of Psychology, University of Sydney, Camperdown NSW 2050, Australia; Lambert Initiative for Cannabinoid Therapeutics, University of Sydney, Camperdown NSW 2050, Australia; Faculty of Science, School of Psychology, University of Sydney, Camperdown NSW 2050, Australia; Edith Collins Centre for Translational Research (Alcohol, Drugs & Toxicology), Royal Prince Alfred Hospital, Sydney Local Health District, Camperdown, NSW 2050, Australia; Specialty of Addiction Medicine, Sydney Medical School, Faculty of Medicine and Health, University of Sydney, Camperdown NSW 2050, Australia; Edith Collins Centre for Translational Research (Alcohol, Drugs & Toxicology), Royal Prince Alfred Hospital, Sydney Local Health District, Camperdown, NSW 2050, Australia; Specialty of Addiction Medicine, Sydney Medical School, Faculty of Medicine and Health, University of Sydney, Camperdown NSW 2050, Australia

**Keywords:** cannabidiol, alcohol use disorder, skin conductance response, high-frequency heart rate variability, risky decision-making

## Abstract

**Aims:**

To examine the effects of cannabidiol (CBD) on psychophysiological responding and risky decision-making in individuals with alcohol use disorder (AUD).

**Methods:**

In a double-blind, placebo-controlled crossover trial, 18 participants with AUD completed the Columbia Card Task (CCT) following administration of CBD and placebo. Skin conductance responses (SCR) and high-frequency heart rate variability (HF-HRV) were assessed as indices of autonomic responding during task performance. Risk-taking was indexed by the number of cards selected on the CCT.

**Results:**

CBD did not significantly affect SCR (*P* = .16, *p*_BH_ = .326) or risky decision-making on the CCT (*P* > .63, *p*_BH_ = 0.67). However, CBD significantly increased HF-HRV (β = 0.53, SE = 0.19, *P* = .005; *p*_BH_ = 0.020). There was no evidence that the relationships between HF-HRV or SCR and risky decision-making differed between CBD and placebo conditions (drug × HF-HRV: *P* = .92; drug × SCR: *P* = .958; both *p*_BH_ = .958).

**Discussion:**

CBD increased HF-HRV but did not significantly alter SCR, risky decision-making, or the relationship between autonomic responses and risky decision-making. These findings suggest that CBD influences autonomic responding during task performance, although the functional significance of this effect remains uncertain. The modest sample size and relatively healthy, non-treatment-seeking sample may have limited the ability to detect broader physiological and behavioural effects.

## Teaser

**Short Summary** This double-blind, placebo-controlled crossover trial examined cannabidiol (CBD) effects on autonomic function and risky decision-making in alcohol use disorder. CBD increased high-frequency heart rate variability, consistent with increased vagally mediated parasympathetic activity, but did not alter skin conductance or risk-taking. The functional and clinical significance of these effects remains uncertain.

## Introduction

Alcohol use disorder (AUD) is the most prevalent substance use disorder globally accounting for 5.1% of the worldwide disease burden (World Health Organization 2018). AUD is defined as a chronic relapsing condition marked by compulsive alcohol seeking and consumption despite detrimental health effects (Haber and Riordan 2021). Diagnostic Statistical Manual (DSM-5; American Psychiatric 2022) criteria for AUD include: unsuccessful attempts to reduce consumption, continued alcohol use despite problems (physical, psychological or social) and using alcohol in situations where this creates more hazard (Haber and Riordan 2021), all of which may reflect impairments in risky decision-making. Persistent, heavy alcohol use leads to neurobiological dysregulation associated with downstream perturbation to behaviour and cognition and a higher propensity for risky decision-making (Ariesen et al. 2023). In fact, high volumes of acute and chronic alcohol consumption have been shown to share a bidirectional causal relationship with risky decision-making (Melugin et al. 2021), with high risk-taking tendency also predicting alcohol relapse (Yuan et al. 2024). Risky decision-making is therefore a potential therapeutic target for pharmacotherapy.

Conventional pharmacotherapy and psychotherapy for AUD show modest results and have variable efficacy (Morley et al. 2021), highlighting the need for novel interventions that can address heterogeneous clinical profiles. Cannabidiol (CBD), a constituent found in high volumes within the *Cannabis sativa* plant, is being investigated for its potential as a pharmacological intervention. Preclinical studies report that CBD reduces drug- and alcohol-motivated behaviours, decreases relapse tendency (Viudez-Martínez et al. 2018), normalizes withdrawal-associated behaviour (Gasparyan et al. 2023), and improves impulsive decision-making following a 7-day dosing regimen in alcohol-dependent animals (Gonzalez-Cuevas et al. 2018). CBD has been shown to modulate serotonergic systems which regulate stress, the reward system and impulsivity (De Gregorio et al. 2019), all of which are central to AUD. Although preclinical literature appears to suggest that CBD may be particularly well suited to manage AUD, clinical literature examining efficacy remains mixed and CBD’s therapeutic mechanisms remain elusive. For example, CBD administration has been associated with reductions in cue-induced craving and decreased activation of reward-related regions such as the nucleus accumbens (Zimmermann et al. 2025). Further, CBD has been demonstrated to decrease anxiety and stress in humans (Masataka 2019). Other research, however, showed no effects of CBD on alcohol use (for 7 days post dosing) or psychophysiological measures (Kirkland et al. 2025). Therefore, further research is needed to better understand the pharmacotherapeutic mechanisms of CBD to determine whether inconsistent findings reflect effects confined to specific clinical subgroups or a generally modest pharmacological impact.

One potential pathway through which CBD may exert differential effects is via modulation of autonomic processes and risky decision-making. Decision-making is closely linked to both the sympathetic and parasympathetic arms of the autonomic nervous system (ANS) (Crone and Van der Molen 2004). These divisions of the ANS may be investigated through measures of heart rate variability (HRV) and skin conductance response (SCR). SCRs, indices of acute sympathetic activation, reflect arousal to stressful or salient stimuli and have been shown to index anticipation of negative or aversive outcomes, thereby shaping attention and guiding decision-making behaviour (Bamshad et al. 2025). HRV, defined as the variation in the interval between heartbeats, reflects ANS function (Mccraty and Shaffer 2015). As a response to stimuli and surroundings, high frequency (HF-) changes in HRV is often interpreted as a marker for executive function processes (Karpyak et al. 2014) with higher HF-HRV reflecting vagally-mediated parasympathetic activity linked to enhanced executive function and autonomic emotional control (Thayer and Siegle 2002). These measures are therefore particularly adept to examining potential pharmacotherapeutic mechanisms in AUD management given persistent alcohol use leads to peripheral nervous system dysfunctions and impaired decision-making which contribute to continued drinking and poorer clinical outcomes.

Therefore, the present study aimed to examine the effect of CBD, relative to placebo, on ANS responses during risky decision-making in individuals with AUD. Based on this evidence, we hypothesized that CBD administration would be associated with increased HF-HRV and decreased SCRs. We further expected that these ANS changes would be associated with reductions in risky decision-making. Such findings could inform future clinical applications of CBD as a tool to support relapse prevention, detoxification and management of AUD-related impairments.

## Materials and methods

### Design

This double-blind, placebo-controlled, crossover trial was conducted over a period of 48 months between 2021 and 2024 at the Royal Prince Alfred Hospital (RPAH) in Sydney, NSW, Australia. The Human Ethics Review Committee of the Sydney Local Health District (X19-0416) approved the trial, and the Sydney Local Health District was a sponsor of the trial. The trial protocol, including design and analytic plans, was previously published (Hurzeler et al. 2024) and the trial was registered in the Clinical Trials Registry (NCT05387148).

### Subjects

Twenty-two non-treatment seeking individuals were recruited to participate, all of whom met the current AUD criteria of the DSM-5 (American Psychiatric 2022). Participants underwent three days of dosing and experimental sessions, per arm. Results from the psychophysiological experiments (day II) are presented in this paper. Participants were recruited through flyers and advertisements at local general practitioners, in newspapers and on websites. RPAH outpatients were also referred through their treating physicians, nurses, and psychologists. In brief participants were eligible for participation if they were aged 18–65 years, satisfied criteria for a diagnosis of AUD, and were presented with a breath alcohol concentration of 0.00 prior to each experimental session. Exclusion criteria included major psychotic disorders, substance use disorders other than nicotine, and medications that could interfere with study outcomes. For full eligibility criteria are described in the published study protocol (Hurzeler et al. 2024).

### Procedure

Participants provided written informed consent after screening and having met eligibility criteria. Post-consent, participants were randomly assigned to one of two treatment regimens: 800 mg CBD (4 × 200 mg gel capsules per day, for 3 days) or matched placebo. A period of at least 18 days (average: 29.2 days ±11.25) was implemented as a ‘washout period’, after which participants would receive the alternative treatment, at the subsequent session. This period was chosen based on the half-life of CBD after chronic administration, defined as 2–5 days (Millar et al. 2018). A minimum 13 day ‘washout period’ was required for effective elimination of CBD from plasma (Mccartney et al. 2023), this washout period was necessitated by our crossover design (see Fig. 1 for the CONSORT flow diagram). The first day of the trial (T1) consisted of the participants being administered their first dose of CBD or placebo and completing baseline sample characteristic questionnaires. During T2, the second day of dosing, the psychophysiological cognitive task (Columbia Card Task [CCT]) was performed, together with questionnaires and the psychophysiological cue reactivity task.

**Figure 1:**
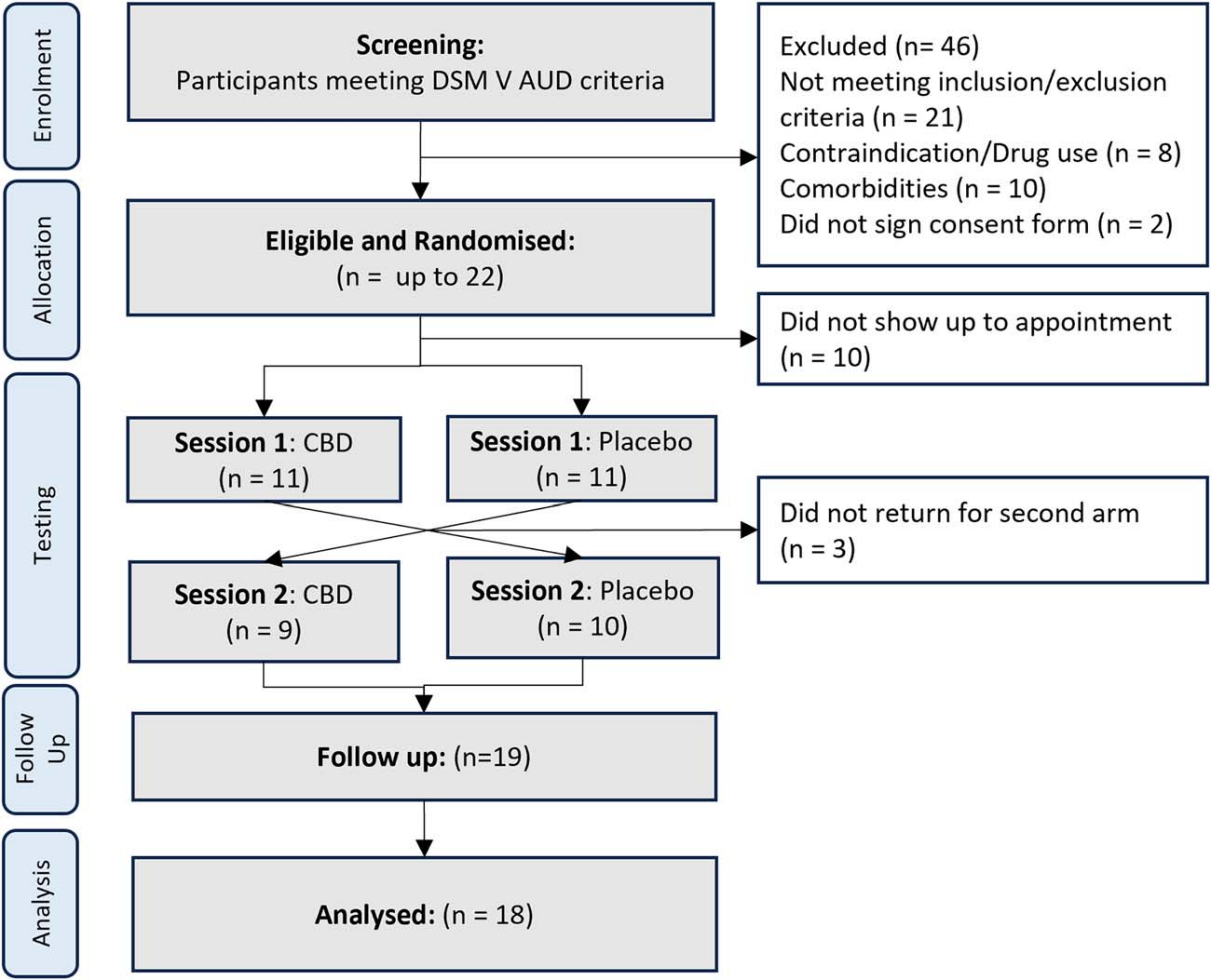
Participant flow diagram showing screening, randomization, crossover allocation to CBD and placebo, follow-up, and inclusion in the final analysis.

### Medication

Both placebo and CBD groups received soft gel capsules weighing 200 mg, identical in appearance, taste and composition (except for the presence of CBD). The CBD group were administered capsules containing medium chain triglyceride (MCT) oil and 200 mg of CBD (manufactured by Linnea Natural Pharma Solutions; Linnea ECS315, Lavertezzo, Switzerland). The placebo group capsules contained only MCT oil, purchased via BOD (Bod ECS100, Double Bay, NSW, Australia). The dosing regimen was followed for 3 days in both the CBD and placebo arm, this schedule read: T1 (Day I): 4 × 200 mg; T2 (Day II): 4 × 200 mg; T3 (Day III): 4 × 200 mg. The medication was administered orally, with water, and under supervision of a research assistant to ensure proper compliance. The daily dose of 800 mg was based on contemporaneous emerging clinical studies as further specified in Hurzeler et al. (2024). The 3-day sub-acute dosing regimen was selected to ensure sustained CBD exposure before experimental testing while maintaining feasibility within the crossover design and limiting participant burden.

### Randomization and allocation concealment

A researcher who was not connected to the trial computer-generated a randomization table using R Studio. Research Electronic Data Capture (REDCap) was utilized by an independent clinical trial pharmacist to randomize participants into one of the two treatment orders, namely CBD first and placebo second: or placebo first and CBD second. All participant data and outcomes were stored in REDCap.

### Measures

#### Questionnaire battery

Eligible participants completed a comprehensive baseline assessment capturing sociodemographic characteristics, alcohol use patterns, and psychopathology. This included measures of alcohol dependence (Alcohol Dependence Scale; ADS), alcohol craving (Penn Alcohol Craving Scale; PACS), mood and stress symptoms (Depression, Anxiety and Stress Scale–21; DASS-21), and insomnia (Insomnia Severity Index; ISI). Psychiatric comorbidities were assessed using the Mini International Neuropsychiatric Interview. For more details see the published study protocol (Hurzeler et al. 2024).

#### Columbia Card Task

The CCT was developed by Figner et al. (2009) to elicit both affective and deliberative decision processes through different task variants (hot, warm, and cold). We used the warm version (Huang et al. 2013), which targets affective decision-making while avoiding the inherent censoring of the hot version. In the warm CCT, 32 cards are presented face down. Participants are informed that certain cards are ‘gain cards’ (make money when turned over), while others are ‘loss cards’ (lose money when turned over, the round ends). Unlike the hot version, which ends immediately upon selecting a loss card, the warm version provides feedback only after the participant has selected all desired cards in each trial, reducing premature termination (Millisecond Software). This allows a more complete index of risk-taking, especially relevant for AUD populations in whom affect-driven decisions are clinically salient (De Groot and Van Strien 2019). Participants’ actions (start new trial, select a card, reveal outcome) were recorded with computer software to the closest millisecond. Consistent with previous work (Lorenz and Ferdinand 2025), the number of cards selected served as our measure of risky decision-making. This has been further verified in Figner et al. (2009), where higher card-turn counts on the CCT significantly correlate with elevated scores on the Adolescent Risk-Taking Scale and self-reported real-world delinquency. In order to mitigate external confounding factors and enhance further measurement sensitivity, participants completed the CCT in a low-stimulus environment isolated from environmental distractions, where researchers refrained from verbal communication once the CCT had commenced.

### Psychophysiological acquisition and transformation

#### Skin conductance

SCRs, in microSiemens (μS), were recorded through MLT117F GSR electrodes placed on the II and III fingers of the non-dominant hand. Eccrine sweat glands are regulated through the ANS’s sympathetic division and are more closely linked with mental processes than thermoregulation (Dawson et al. 2011). This specific type of sweating from volar surfaces, also known as emotional sweating, occurs during decision-making processes (Kluwe-Schiavon et al. 2015). Signals were amplified using a FE116 GSR amplifier and sampled at 1000 Hz through a PowerLab 8/25 system (LabChart Pro 7.3.7). Low- and high-pass filters were applied to remove high-frequency noise and tonic shifts, respectively. We isolated decision-making blocks (mean interval 2807 ms) using a marker detection function. SCR amplitude was quantified as the area bound by the curve of the filtered signal divided by block duration resulting in units of μS/s (Naqvi and Bechara 2006). This area bound by the curve metric captures both amplitude and temporal dynamics (Figner and Murphy 2011).

#### Heart-rate variability

An electrocardiogram (ECG) was collected continuously during the CCT using a three-lead Ag/AgCl configuration (non-dominant wrist as ground; leads above the cubital fossa on each arm). Signals were amplified using an ML408 Dual Bioamp/Stimulator and sampled at 1000 Hz (PowerLab 8/25; LabChart Pro 7.3.7; ADInstruments, 2012). Artefacts were identified automatically and manually, then corrected via interpolation in Kubios HRV. The InterBeat Intervals (IBIs) were extracted from the ECG signal. These IBIs constitute the distance between two consecutive heartbeats, measured as the distance between two R-peaks on the ECG, otherwise known as HRV. A 5-min neutral-stimulus video served as baseline. The CCT was divided into three consecutive 5-min blocks; HF-HRV was averaged across the blocks and calculated using Fast Fourier Transform based spectral analysis in the 0.15–0.40 Hz band. HF-HRV, measured in ms^2^, was analysed in normalized units.

### Statistical analysis

Generalized Estimating Equations (GEEs) were used with an exchangeable correlation structure to test the effects of CBD on SCRs, HF-HRV and risky decision-making. SCR and HF-HRV were log transformed to reduce skew. Age, sex, and drinking on the previous day (DDB; drinking day before) were included as covariates due to their known effects on autonomic function (Koskinen et al. 1994, Stewart and Pihl 1994). Session order and carryover were modelled using Crosscarry’s CreateCarry package (Cruz et al. 2026).

Risky decision-making was examined using two complementary approaches: (i) an SCR-linked analysis modelling the number of cards selected at each of 63 trials, and (ii) an HF-HRV-linked analysis modelling the mean number of cards selected across the session. Separate GEE interaction models examined whether the association between physiological responses and risky decision-making differed by drug condition, incorporating either a drug × SCR or drug × HF-HRV interaction term.

To account for multiple comparisons, Benjamini–Hochberg false discovery rate correction was applied separately to two families of primary hypothesis tests: (i) main drug effects across SCR, HF-HRV and the two risky decision-making analyses, and (ii) drug × physiological response interaction effects. Both unadjusted and BH-adjusted *P*-values were reported, with statistical significance determined at an adjusted *P* < .05.

## Results

Twenty-two participants completed at least one arm of the trial; three participants did not complete both sessions, and one of the participant’s SCR data was lost due to equipment malfunction during the research session.

### Sample characteristics

Table 1 depicts the sample characteristics, with the mean age of the participants being 30.33 (± 12.72). These sample characteristics were collected at their baseline appointment (screening). The ADS score models the degree of AUD severity in our participants, which was 16.17 (± 7.97) on average. It is important to note that our population consisted of non-treatment seeking individuals, which is reflected in this score. Tobacco and anti-depressant use within the past 28 days was present in 5 out of 18 and 6 out of 18 participants, respectively. Comorbidities were reported by 6 out of 18 participants. Of these, five met criteria for an anxiety disorder (current GAD, social phobia, or panic disorder), while one met criteria for current major depressive episode. Anxiety disorders were grouped due to their shared clinical features. On average, 7.76 (± 5.87) standard drinks were consumed by participants per day, who had experienced an average of 8.94 (± 9.79) years of problematic drinking. The average ISI score of our population was 8.56 (± 6.56) with the average PACS score being 12.35 (± 5.35). On the DASS our participants scored, on average, 7.34 (± 6.28) on the anxiety subscale (normal severity); 12 (± 10.42) on the depression subscale (mild severity) and 14.34 (± 9.64) on the stress subscale (normal severity).

**Table 1:** Characteristics of the participants at baseline.

Sample characteristics	*n*	%	M	SD
Gender (Female)	12	66		
Age (years)			30.33	12.72
SDD (standard drinks per day)			7.76	5.87
Years since alcohol-related problems began			8.94	9.79
ADS score			16.17	7.97
PACS craving score			12.35	5.35
ISI score			8.56	6.56
Comorbidities	6	33		
Anti-depressant use	6	33		
Tobacco use	5	28		
DASS				
Anxiety subscale			7.34	6.28
Depression subscale			12	10.42
Stress subscale			14.34	9.64

Note. *N* = 18. Unless otherwise specified, values are reported as means with respective standard deviations. SDD = standard drinks per day over the previous 28 days.

### Skin conductance response

The SCR outcome was not significantly associated with CBD (β = −0.64, SE = 0.45, *P* = .16, *p*_BH_ = 0.33), as seen in Fig. 2*,* with full model details in Supplementary Table S1. Males had higher log SCR values than females (β = 0.81, SE = 0.27, *P* < .01). DDB had no significant effect on SCR outcome, and neither did age, session or any carryover effects (*P* > .05).

**Figure 2:**
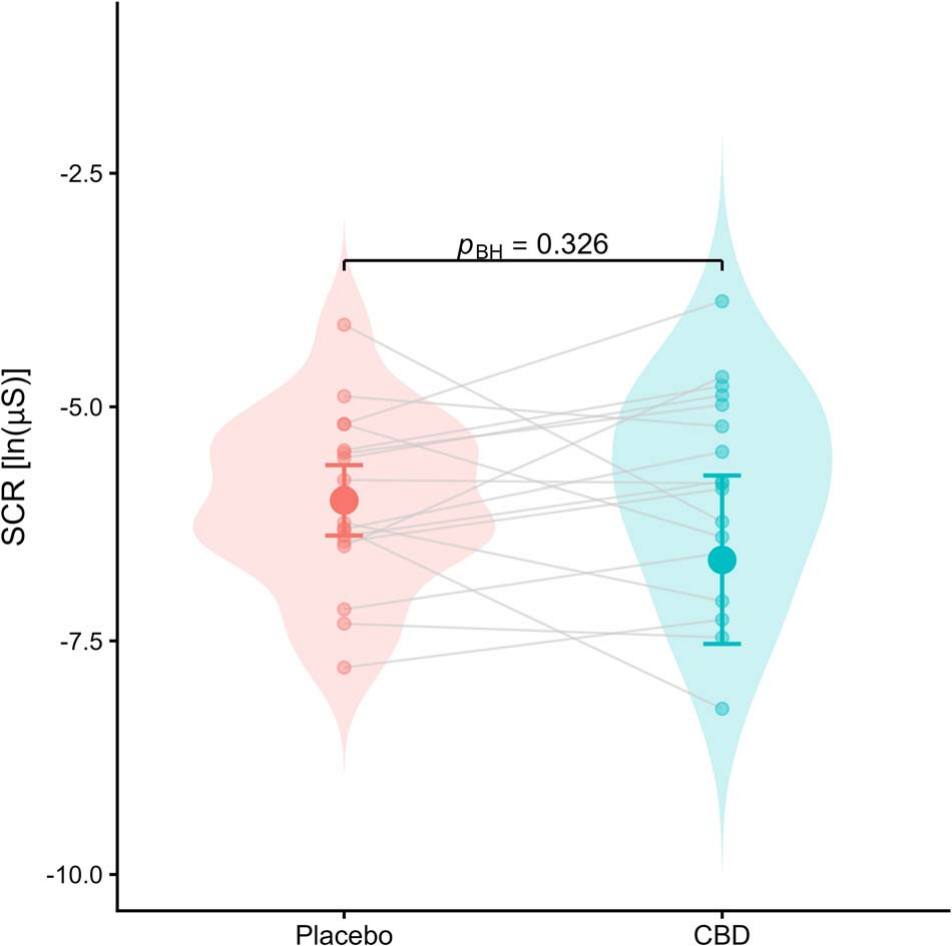
Mean log-transformed skin conductance responses (SCRs) under placebo and CBD conditions [ln(μS)], showing individual observations and within-participant connections, distributions, and Gaussian GEE-estimated marginal means with 95% CIs; the drug effect was non-significant (*P* = .163; *p*_BH_ = .326), with the BH-adjusted *P*-value displayed.

### High-frequency heart rate variability

CBD administration had a statistically significant impact on higher HF-HRV outcomes (β = 0.53, SE = 0.19, *P* = .005, *p*_BH_ = 0.02), as seen in Fig. 3, with full model details in Supplementary Table S2. HF-HRV was lower throughout the task phase than baseline (β = −0.36, SE = 0.08, *P* < .01). Session had a significant effect on our HF-HRV outcome (β = −0.41, SE = 0.19, *P* = .032), with the second session having lower HF-HRV outcomes. Additionally, there was a carryover effect (β = 0.71, SE = 0.31, *P* = .024), with higher HF-HRV outcomes in the placebo setting post CBD exposure. Sex, age, and DDB status did not significantly affect HF-HRV (*P* > .10).

**Figure 3:**
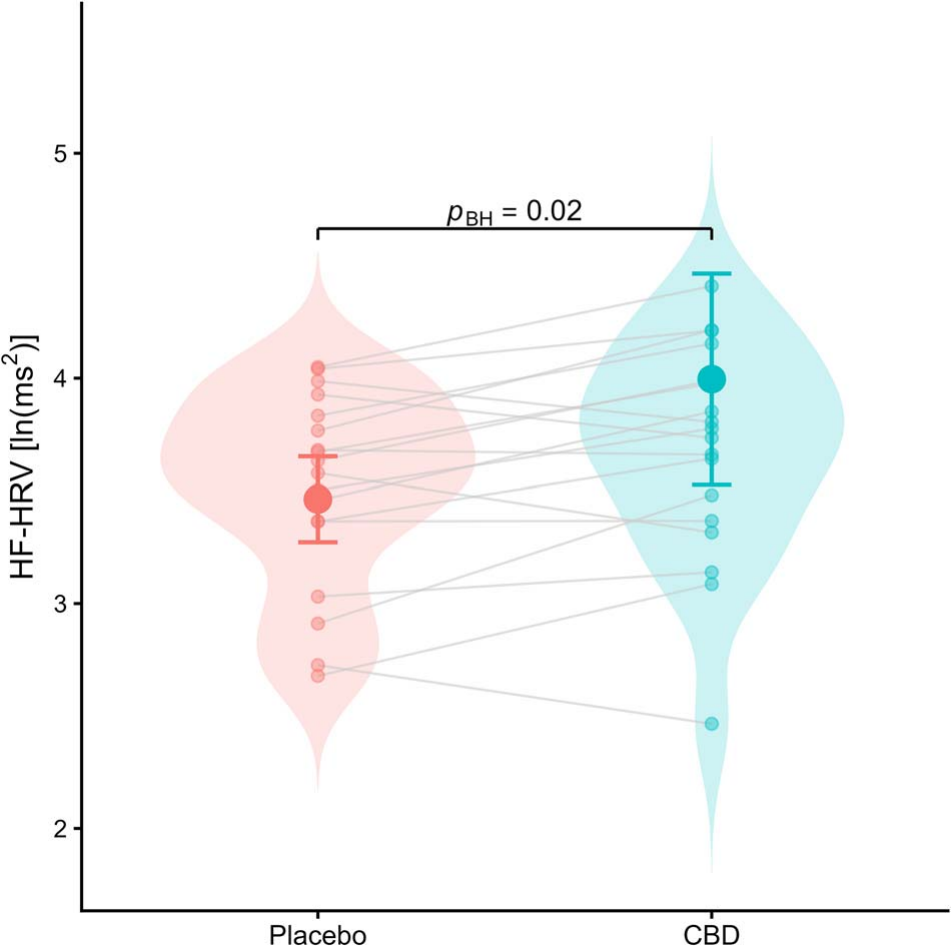
Mean log-transformed high-frequency heart rate variability (HF-HRV) under placebo and CBD conditions [ln(ms²)], showing individual observations and within-participant connections, distributions, and Gaussian GEE-estimated marginal means with 95% CIs; the drug effect was significant (*P* = .005; *p*_BH_ = .020), with the BH-adjusted *P*-value displayed.

### Risky decision-making

Analysis I: There was no evidence that CBD significantly impacted risky decision-making (β = 1.1, SE = 2.61, *P* = .67, *p*_BH_ = 0.67). Sex was a significant predictor for risky decision-making, with males choosing more cards than females (β = 6.22, SE = 2.41, *P* = .01).

In analysis II, CBD had no significant impact on the number of cards chosen (β = 1.2, SE = 2.53, *P* = .63, *p*_BH_ = 0.67). Sex did have a significant effect on the number of cards chosen, with male participants choosing more cards than females (β = 6.17 SE = 2.50, *P* = .014). Age, session, DDB, carryover effects showed no significant effects on the number of cards chosen (*P* > .295 for all).

These analyses are visualized in Fig. 4, with full model details in Supplementary Tables S3 and S4.

**Figure 4:**
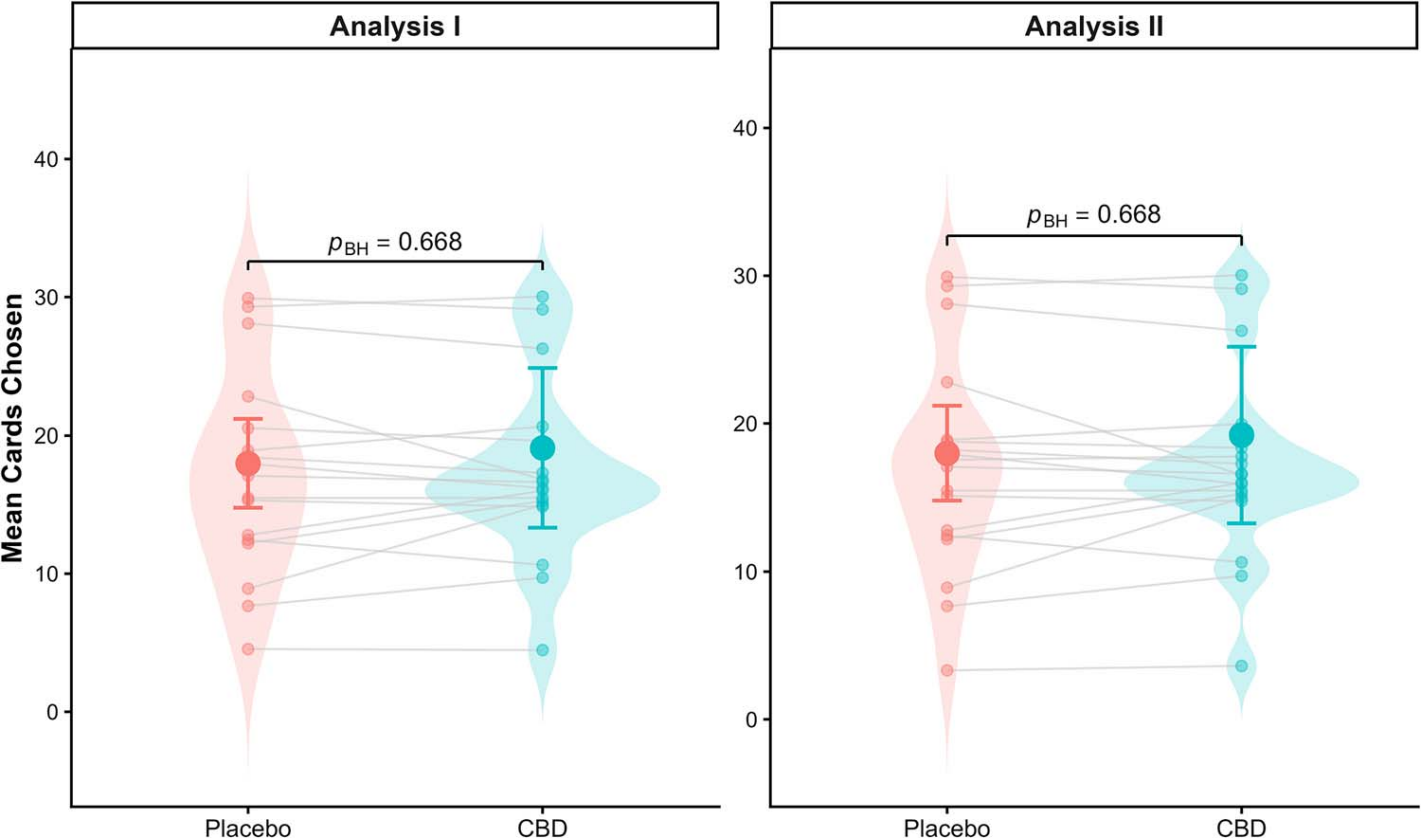
Mean number of cards selected under placebo and CBD conditions across two risky decision-making analyses (analysis I: trial-level SCR data; analysis II: session-level HF-HRV data with one additional participant), showing individual observations, within-participant connections, distributions, and Gaussian GEE-estimated marginal means with 95% CIs; drug effects were non-significant in both analyses (analysis I: *P* = .668; analysis II: *P* = .628; both *p*_BH_ = .668), with BH-adjusted *P*-values displayed.

### Interaction models

Separate GEE models examined whether HF-HRV and SCR were associated with risky decision-making and whether these associations differed between CBD and placebo. There was no significant drug × HF-HRV interaction on mean cards chosen (β = 0.14, SE = 1.35, *P* = .92), which remained non-significant following Benjamini–Hochberg correction (*p*_BH_ = .958). Similarly, there was no significant drug × SCR interaction (β = −0.03, SE = 0.55, *P* = .958; *p*_BH_ = .958). Full model details in Supplementary Tables S5 and S6.

## Discussion

The current study examined whether CBD modulates autonomic responses and whether these changes are associated with alterations in risky decision-making, with the aim of elucidating potential mechanisms underlying its use in AUD. CBD administration was not associated with significant changes in SCR or the number of cards selected, indicating no observable effect on sympathetic arousal or risk-taking. However, HF-HRV was significantly elevated during CBD sessions, consistent with previous findings (Williams et al. 2021) and replicating this effect within the same sample across a distinct task (Hurzeler et al. 2025b). We further examined whether CBD altered the relationship between physiological responses and risky decision-making. Neither the drug × SCR nor drug × HF-HRV interaction significantly predicted the number of cards chosen, suggesting that although CBD increased HF-HRV, this physiological effect was not accompanied by a detectable change in the relationship between autonomic responses and risky decision-making.

The absence of modulation of sympathetic arousal and risky decision-making contrasts with our hypothesis and does not support the originally proposed mechanism whereby CBD influences decision-making and subsequently seeking behaviours via broad autonomic modulation. However, when considered with respect to existing literature, these findings provide insight into alternative mechanisms through which CBD may be relevant to AUD. Specifically, the present results replicate previous findings of increased HF-HRV following CBD administration, without corresponding changes in phasic sympathetic arousal indexed by SCR. Higher HF-HRV is commonly interpreted as reflecting greater vagally mediated parasympathetic activity; however, HF-HRV is influenced by multiple physiological and contextual factors and should not be considered a direct measure of stress regulation. Moreover, the absence of corresponding changes in risky decision-making or significant physiological–behavioural interactions limits conclusions regarding the functional significance of the observed HF-HRV effect.

This interpretation is consistent with previous literature examining CBD in AUD and other substance use disorder. For example, neuroimaging studies demonstrate that CBD attenuates activity in regions such as the amygdala and precuneus, which are implicated in threat/emotional salience and rumination (Pfisterer et al. 2026, Hurzeler et al. 2025a). Furthermore, no recent publications that we are aware of indicate changes to cognition when comparing CBD to placebo in a clinical sample, however various publications cite changes to anxiety and craving. For example, in the current sample, CBD was associated with increased parasympathetic activity during alcohol cue exposure, alongside reductions in cue-elicited anxiety and post-exposure alcohol craving, while cognitive task results remained unmodulated, supporting a stress-regulatory rather than cognitive-enhancement mechanism of action (Hurzeler et al. 2025b). Similar results have also been observed in individuals experiencing AUD by which acute CBD administration reduced cue-induced craving and anxiety, as well as physiological stress markers such as heart rate and salivary cortisol without changes observed in cognitive performance (Hurd et al. 2019). In this context, CBD may reduce the affective and motivational impact of alcohol-related cues by dampening limbic reactivity and internally driven craving processes, rather than directly altering decision-making behaviour.

Taken together with previous findings, the observed increase in HF-HRV raises the possibility that CBD may influence stress-related physiological processes rather than directly affecting cognitive control or decision-making. However, the present study did not directly assess stress regulation, and the functional significance of the HF-HRV effect therefore remains uncertain. This interpretation of the results would suggest that CBD may be suited for individuals with AUD and comorbid anxiety, or for those whose alcohol use is driven by negative reinforcement processes such as tension reduction. In these populations, targeting stress reactivity and autonomic dysregulation may reduce vulnerability to cue-induced craving and relapse, even in the absence of measurable changes in cognitive task performance. However, as the present study evaluated a non-treatment seeking population with lowered clinical levels of autonomic dysregulation or substance dependence, these therapeutic implications remain speculative until validated within treatment-seeking clinical samples.

The results presented here should be appraised with respect to various strengths and limitations. Firstly, this is to our knowledge the first study examining the effect of CBD compared to placebo on the relationship between psychophysiological modulation and its direct relationship with risky-decision making. We employed a rigorous double-blind crossover design and GEEs, to account for within-subject effects and potential carryover effects. However, blinding integrity was not formally assessed, as participant and investigator guesses regarding treatment allocation were not collected. Consequently, the success of blinding cannot be determined. The sample comprised non-treatment seeking individuals with relatively mild AUD, which may have masked cognitive or sympathetic nervous system effects. In addition, the modest sample size, and exclusion of one participant from the SCR analysis due to technical issues, limited statistical power, particularly for detecting interaction effects. Accordingly, the non-significant effects observed for SCR, risky decision-making, and the physiological–behavioural interaction analyses should not be interpreted as definitive evidence of an absence of effect. The CCT demonstrates moderate test–retest reliability across repeated administrations (*r* = .57; Buelow and Barnhart 2018), although the potential influence of repeated task exposure on the observed carryover effects cannot be excluded. Furthermore, the CCT captures a specific laboratory-based dimension of risk-taking and may not fully represent the complex affective, social and contextual determinants of alcohol-related decision-making in everyday settings. The three-day dosing regimen was designed to assess short-term autonomic and psychophysiological responses; longer treatment durations may be required to detect cognitive adaptations. Finally, although the washout period was selected to exceed the expected period of systemic CBD persistence, a significant carryover effect was observed for HF-HRV. This may reflect residual period-related effects or longer-lasting physiological effects of CBD that persisted beyond the treatment period. Accordingly, the HF-HRV treatment effect should be interpreted with caution, and future studies should examine the duration of these effects using longer washout periods.

In conclusion, CBD, compared with placebo, increased HF-HRV without measurable changes in phasic sympathetic arousal or risk-taking behaviour. Although increased HF-HRV is consistent with greater vagally mediated parasympathetic activity, its functional significance remains uncertain in the absence of corresponding behavioural effects. Future research should examine whether CBD-induced changes in parasympathetic function modulate other domains of executive functioning and whether these effects are associated with meaningful clinical outcomes, particularly in more severe or treatment-seeking populations. Stratification by comorbid anxiety and individual differences in stress responsivity may further clarify the clinical relevance of these effects.

## Supplementary Material

Supplementary_Materials_260901_CLEAN_agag068

## Data Availability

The data that support the findings of this study are available from the corresponding author upon request.
