# Supplementary material for: Effects of cannabidiol on risky decision-making and autonomic function in alcohol use disorder
Source: Alcohol Alcohol. 2026 Oct 14;61(6):agag068. doi: 10.1093/alcalc/agag068 (PMC13665463; doi:10.1093/alcalc/agag068)
Supplement: Supplementary file 1 [file supplementary_materials_260901_clean_agag068.docx]

# Supplementary Materials

## Methods

### Columbia Card Task

The warm version presents 32 cards face down. Participants are informed that certain cards are ‘gain cards’ (make money when turned over), while others are ‘loss cards’ (lose money when turned over, the round ends). They are also kept informed at all times about the number of ‘loss cards’; the amount of money they can make (in total) from each ‘gain card’ and the amount of money that they will lose if a loss card is turned over.

In the warm version, participants only get to see the result of their choices after they have picked all the cards that they want to turn over, per round (Millisecond Software, n.d.).

It is important to note that the results of the cards rely on rigged feedback, meaning that the last cards turned over will always be the ‘loss cards’. The CCT is programmed to have 54 rounds where the participant does not lose, with only the remaining 9 rounds being random.

Every time a participant chose to start a new game, select a card or to see the results of the round, these actions were recorded with computer software to the closest millisecond.

As detailed in de Groot & van Strien (2019) there are certain benefits to using the warm CCT over the hot version. Just like the warm version, the hot version of the CCT also focuses on affective processes. The data from the hot CCT is inherently censored, which occurs because a round is automatically ended after encountering a loss card, even if the person would have otherwise taken more risk. The delayed feedback in the warm version avoids this inherent censoring. Figner et al. (2009) state that older research on risky decision making was often concentrated on deliberative processes, through the cold version of the CCT.

## Data transformation

### HF-HRV

A three-lead configuration of disposable electrodes (Ag/AgCl) was used to record the initial ECG signal. The electrodes were placed on the patient's non-dominant wrist (which served as a ground electrode) and above the cubital fossa on each arm. Signal amplification was performed using an ML408 Dual Bioamp/Stimulator (ADInstruments; Bella Vista, NSW, Australia), sampled at 1,000 Hz using a PowerLab 8/25 System (ADInstruments; Sydney, Australia) connected to a PC running LabChart Pro 7.3.7 software (ADInstruments, 2012).

Sections containing ECG artefacts were identified both automatically and through manual inspection, and subsequently corrected through interpolation, with use of Kubios HRV software.

The InterBeat Intervals (IBIs) were extracted from the ECG signal. These IBIs constitute the distance between two consecutive heartbeats, measured as the distance between two R-peaks on the ECG.

Participants were instructed to watch a 5-minute video with neutral stimuli, the ECG data during this period was used as a baseline. We were able to split the CCT up into 3 5-minute blocks, of which we took the mean of these 3 blocks as our HF-HRV outcome, measured in normalised units. The time between these 5 minute blocks was 1 second, with the exception of one trial needing an 80 second interval between two blocks due to technical limitations. HF-HRV was calculated using spectral analysis in the 0.15–0.40 Hz frequency range via Fast Fourier Transformation. The HF-HRV within these three blocks was compared to the baseline block, providing robust data quality. A contrast variable was coded to effectively compare HF-HRV to baseline measurements.

### SCR

SCR signals were measured through MLT117F GSR electrodes, placed on the II and III fingers of the non-dominant hand.

The SCRs were amplified using a FE116 GSR amplifier connected to a PC operating LabChart Pro 7.3.7. software, via a PowerLab 8/25 system samping at 1,000 Hz (Hurzeler et al., 2024).

Both a low-pass filter and a high-pass filter were applied to our raw signal, to eliminate high-frequency noise and tonic changes, respectively. SCRs are chosen above Skin Conductance Level (SCL) measurements because, unlike the SCRs, the SCL is thought to be a measure of a more general level of arousal rather than being directly tied to stimuli (Figner et al. 2011).

We studied the area bounded by the curve for the filtered SCR signal, and specifically isolated decision-making blocks. The time between our blocks was 2807 ms, in line with literature (Dawson et al., 2011a). Using a marker detection function, detecting the begin and end points of the blocks, the integral of the absolute area bounded by the curve was measured. This area bound by the curve was then divided by the length of the decision-making interval, in seconds, resulting in units of µS/s (Naqvi & Bechara, 2006). According to (Figner & Murphy, 2011) this area bound by the curve is a more valid indicator for automated data analysis, capturing both the amplitude and the temporal properties of an SCR.

## Generalised Estimated Equations Results

### Table S1: Generalized Estimating Equations (GEE) results: log-transformed skin conductance responses (SCR)

| ***Term*** | ***Estimate*** | ***Std. Error*** | ***Statistic*** | ***p-value*** |
| --- | --- | --- | --- | --- |
| *(Intercept)* | -6.429 | 0.528 | 148.5 | 0 |
| *Drug 1* | -0.636 | 0.456 | 1.95 | 0.163 |
| *Sex (male)* | 0.808 | 0.269 | 9.05 | 0.003* |
| *Age* | 0.013 | 0.009 | 2.04 | 0.153 |
| *DDB* | -0.4 | 0.345 | 1.34 | 0.247 |
| *Carryover effect* | -1.292 | 0.697 | 3.44 | 0.064 |
| *Session* | 0.955 | 0.498 | 3.69 | 0.055 |

*Note. Results from generalized estimating equations (GEE) examining the effects of drug condition (CBD vs placebo) and covariates on log-transformed SCR. Sex is coded as male relative to female (reference). Age and DDB were included as continuous covariates. Carryover effect and session were included to account for the cross-over design. Estimates (β), standard errors (SE), Wald statistics, and p-values are reported. *p < .05. The model included the following variables:*

- *Session 1: Session order effect.*
- *Carry 2: Carryover effect (AB/BA).*
- *Drug 1: Treatment with cannabidiol (CBD).*
- *DDB: Drinks consumed in the previous 24 hours.*

*The GEE model was specified as follows:*

*SCR ∼ Drug group (CBD) + Sex + Age + Drinks in Previous 24 Hours + Carryover Effect + Session Order*

*An exchangeable working correlation structure was used to estimate treatment effects, controlling for session order and carryover effects.*

### Table S2: Generalized Estimating Equations (GEE) Results: mean log transformed High Frequency Heart rate variability (HF-HRV)

| ***Term*** | ***Estimate*** | ***Std. Error*** | ***Statistic*** | ***p-value*** |
| --- | --- | --- | --- | --- |
| *(Intercept)* | 3.229 | 0.34 | 90.41 | 0 |
| *CBD* | 0.533 | 0.192 | 7.726 | 0.005* |
| *Sex (male)* | -0.397 | 0.249 | 2.533 | 0.111 |
| *Age* | 0.003 | 0.005 | 0.547 | 0.459 |
| *DDB* | -0.072 | 0.123 | 0.34 | 0.56 |
| *Carryover effect* | 0.708 | 0.313 | 5.126 | 0.024* |
| *Session* | -0.408 | 0.19 | 4.606 | 0.032* |
| *Contrast* | -0.357 | 0.079 | 20.59 | 0.000* |

*Note. Results from generalized estimating equations (GEE) examining the effects of drug condition (CBD vs placebo) and covariates on log-transformed HF-HRV. Sex is coded as male relative to female (reference). Age and DDB were included as continuous covariates. Carryover effect and session were included to account for the cross-over design. Estimates (β), standard errors (SE), Wald statistics, and p-values are reported. *p < .05. The model included the following variables:*

- *Session 1: Session order effect.*
- *Carry 2: Carryover effect (AB/BA).*
- *Drug 1: Treatment with cannabidiol (CBD).*
- *DDB: Drinks consumed in the previous 24 hours.*
- *Contrast: Task block vs baseline*

*The GEE model was specified as follows:*

*HF-HRV ∼ Drug group (CBD) + Age + Sex + Drinks in Previous 24 Hours + Carryover Effect + Session Order+ Contrast*

*An exchangeable working correlation structure was used to estimate treatment effects, controlling for session order and carryover effects*

### Table S3: Generalized Estimating Equations (GEE) Results: Analysis 1 – cards per round

| ***Term*** | ***Estimate*** | ***Std. Error*** | ***Statistic*** | ***p-value*** |
| --- | --- | --- | --- | --- |
| *(Intercept)* | 14.955 | 4.302 | 12.085 | 0.001 |
| *CBD* | 1.119 | 2.609 | 0.184 | 0.668 |
| *Sex (male)* | 6.043 | 2.463 | 6.017 | 0.014* |
| *Age* | -0.009 | 0.091 | 0.009 | 0.923 |
| *DDB* | -1.231 | 1.15 | 1.145 | 0.285 |
| *Carryover effect* | 2.36 | 5.038 | 0.219 | 0.639 |
| *Session* | -0.572 | 2.473 | 0.054 | 0.817 |

*Note. Results from generalized estimating equations (GEE) examining the effects of drug condition (CBD vs placebo) and covariates on cards selected per round. Sex is coded as male relative to female (reference). Age and DDB were included as continuous covariates. Carryover effect and session were included to account for the cross-over design. Estimates (β), standard errors (SE), Wald statistics, and p-values are reported. *p < .05. The model included the following variables:*

- *Session 1: Session order effect.*
- *Carry 2: Carryover effect (AB/BA).*
- *Drug 1: Treatment with cannabidiol (CBD).*
- *DDB: Drinks consumed in the previous 24 hours.*
- *Contrast: Task block vs baseline*

*The GEE model was specified as follows:*

*Cards selected per round ∼ Drug group (CBD) + Sex + Age + Drinks in Previous 24 Hours + Carryover Effect + Session Order*

*An exchangeable working correlation structure was used to estimate treatment effects, controlling for session order and carryover effects*

### Table S4: Generalized Estimating Equations (GEE) Results: Analysis 2 - (Mean) Number of cards chosen ~ Drug:

| ***Term*** | ***Estimate*** | ***Std. Error*** | ***Statistic*** | ***p-value*** |
| --- | --- | --- | --- | --- |
| *(Intercept)* | 16.262 | 3.851 | 17.828 | 0 |
| *CBD* | 1.225 | 2.529 | 0.235 | 0.628 |
| *Sex (male)* | 6.172 | 2.503 | 6.082 | 0.014* |
| *Age* | -0.057 | 0.091 | 0.386 | 0.534 |
| *DDB* | -1.222 | 1.168 | 1.095 | 0.295 |
| *Carryover effect* | 2.647 | 4.887 | 0.293 | 0.588 |
| *Session* | -0.733 | 2.382 | 0.095 | 0.758 |

Note. Results from generalized estimating equations (GEE) examining the effects of drug condition (CBD vs placebo) and covariates on mean number of cards chosen throughout task. Sex is coded as male relative to female (reference). Age and DDB were included as continuous covariates. Carryover effect and session were included to account for the cross-over design. Estimates (β), standard errors (SE), Wald statistics, and p-values are reported. *p < .05. The model included the following variables:

- Session 1: Session order effect.
- Carry 2: Carryover effect (AB/BA).
- Drug 1: Treatment with cannabidiol (CBD).
- DDB: Drinks consumed in the previous 24 hours.

The GEE model was specified as follows:

Mean number of cards chosen ∼ Session Order + Carryover Effect + Drug group (CBD) + Drinks in Previous 24 Hours

An exchangeable working correlation structure was used to estimate treatment effects, controlling for session order and carryover effects

### Table S5: Generalized Estimating Equations (GEE) Results: Interaction between HF-HRV and Drug on Mean Number of Cards Chosen

| ***Term*** | ***Estimate*** | ***Std. Error*** | ***Statistic*** | ***p-value*** |
| --- | --- | --- | --- | --- |
| *Intercept* | 16.1333 | 3.8628 | 17.44 | 0 |
| *CBD* | 1.4034 | 2.4634 | 0.32 | 0.569 |
| *HF-HRV* | -0.6143 | 1.736 | 0.13 | 0.723 |
| *Sex (male)* | 6.051 | 2.6471 | 5.23 | 0.022* |
| *Age* | -0.0612 | 0.0859 | 0.51 | 0.477 |
| *DDB* | -1.2441 | 1.2303 | 1.02 | 0.312 |
| *Carryover effect* | 3.0744 | 4.7845 | 0.41 | 0.52 |
| *Session* | -0.8422 | 2.3205 | 0.13 | 0.717 |
| *CBD*HF-HRV* | 0.1351 | 1.3506 | 0.01 | 0.92 |

**Note.** Results from generalized estimating equations (GEE) examining whether the association between HF-HRV during the CCT and mean number of cards chosen differed between CBD and placebo. HF-HRV represents HRV measured during the active CCT period. Sex is coded as male relative to female (reference). Age and DDB were included as continuous covariates. Carryover effect and session were included to account for the cross-over design. Estimates (β), standard errors (SE), Wald statistics, and *p*-values are reported. The CBD * HF-HRV interaction remained non-significant following Benjamini–Hochberg correction (*p*BH = .958). **p* < .05.

The model included the following variables:

- Session 1: Session order effect.
- Carry 2: Carryover effect (AB/BA).
- Drug 1: Treatment with cannabidiol (CBD).
- DDB: Drinks consumed in the previous 24 hours.

The GEE model was specified as:

Mean number of cards chosen ∼ CBD*HF-HRV + Sex + Age + DDB + Carryover Effect + Session

An exchangeable working correlation structure was used to account for repeated observations within participants.

### Table S6: Generalized Estimating Equations (GEE) Results: Interaction between SCR and Drug on Mean Number of Cards Chosen

| ***Term*** | ***Estimate*** | ***Std. Error*** | ***Statistic*** | ***p-value*** |
| --- | --- | --- | --- | --- |
| *Intercept* | 11.023 | 6.717 | 2.693 | 0.101 |
| *CBD* | 0.547 | 4.577 | 0.014 | 0.905 |
| *SCR* | −0.812 | 0.688 | 1.394 | 0.238 |
| *Sex (male)* | 6.855 | 2.652 | 6.680 | 0.010* |
| *Age* | −0.046 | 0.099 | 0.219 | 0.640 |
| *DDB* | −1.573 | 1.176 | 1.790 | 0.181 |
| *Carryover effect* | 1.571 | 4.933 | 0.101 | 0.750 |
| *Session* | 0.091 | 2.445 | 0.001 | 0.970 |
| *CBD*SCR* | −0.029 | 0.547 | 0.003 | 0.958 |

**Note.** Results from generalized estimating equations (GEE) examining whether the association between skin conductance response (SCR) and mean number of cards chosen differed between CBD and placebo. Sex is coded as male relative to female (reference). Age and DDB were included as continuous covariates. Carryover effect and session were included to account for the cross-over design. Estimates (β), standard errors (SE), Wald statistics, and *p*-values are reported. The CBD × SCR interaction remained non-significant following Benjamini–Hochberg correction (*p*BH = .958). **p* < .05.

The model included the following variables:

- Session 1: Session order effect.
- Carry 2: Carryover effect (AB/BA).
- Drug 1: Treatment with cannabidiol (CBD).
- DDB: Drinks consumed in the previous 24 hours.

The GEE model was specified as:

Mean number of cards chosen ∼ CBD*SCR + Sex + Age + DDB + Carryover Effect + Session

An exchangeable working correlation structure was used to account for repeated observations within participants.
